# Trends and correlates of cystic echinococcosis in Chile: 2001–2012

**DOI:** 10.1371/journal.pntd.0005911

**Published:** 2017-09-15

**Authors:** Soledad Colombe, Eri Togami, Fkadu Gelaw, Marina Antillon, Rodrigo Fuentes, Daniel Martin Weinberger

**Affiliations:** 1 Department of Epidemiology of Microbial Diseases, Yale School of Public Health, New Haven, Connecticut, United States of America; 2 Facultad de Medicina, Clínica Alemana Universidad del Desarrollo, Santiago, Chile; Negrar Hospital, ITALY

## Abstract

Echinococcosis is a neglected zoonotic disease affecting over 1 million people worldwide at any given time. It is the leading cause of hospital admissions for parasitic diseases in Chile. We conducted a retrospective investigation of hospitalized cases to describe the epidemiological trends of echinococcosis in Chile. We also examined the potential environmental risk factors for echinococcosis hospitalization rates. Through nation-wide hospital discharge data, a total of 11,516 hospitalized patients with cystic echinococcosis were identified between January 2001 and December 2012. The mean age of hospitalization was 40 years, with notable gender difference in pediatric patients. The hospitalization rate was found to be overall steadily decreasing from 2001 (7.02 per 100,000) to 2012 (4.53 per 100,000) with a 5% decrease per year (rate ratio = 0.95 [95% CI: 0.94, 0.96]). The hospitalization rate was higher in the south of Chile compared to the north. Goat density and intermediate precipitation were found to be significantly positively associated with the hospitalization rate while annual average temperature was found to be significantly negatively associated with the hospitalization rate. Findings of this study indicate that echinococcosis is still an important public health burden in Chile related to interaction with livestock and climate. Efforts should be placed on targeted prevention measures for farmers and raising awareness of echinococcosis among health care workers.

## Author summary

Humans are infected by many types of parasites that originate from animals. Cystic echinococcosis (CE) is a tapeworm infection that affects over 1 million people over the world at any given time, with symptoms such as abdominal pain, chest pain, vomiting, and allergic reactions. The lifecycle of this parasite is maintained between dogs and livestock, and humans are ‘accidentally’ infected through ingesting parasite eggs. This makes the control of CE difficult for public health practitioners. Attempts have been made to better understand which animals or environmental factors increase or decrease the number of human CE infections, but mainly with data from only one point in time. In this study, we studied the trends of this disease in Chile, a country with many cases of CE, over a 12-year period. Then, using a statistical model, we examined the risk factors related to animals and the environment that contribute to a higher rate of CE hospitalizations. We found that higher goat density, intermediate precipitation level and low temperatures are important factors associated with an increase in hospitalization rates related to CE in Chile. This new knowledge contributes to a better overall understanding of the trends and risk factors of CE, which will help to implement targeted prevention and control strategies in regions where the disease is common.

## Introduction

Echinococcosis is a neglected zoonotic disease, which affects over 1 million people worldwide at any given time and the loss of 1 to 3 million disability-adjusted life years annually [1]. It is caused by parasitic tapeworms of the genus *Echinococcus*. The two species of clinical and public health importance are *E*. *granulosus*, related to cystic echinococcosis (CE), and *E*. *multilocularis*, related to alveolar echinococcosis (AE) [1–2]. CE affects livestock production, and it is estimated that CE treatment in humans and losses in livestock cost the global economy 3 billion USD every year [1]. Although the cyst of the parasite is slow-growing in the human body, infected people may face debilitating and life-threatening symptoms such as abdominal or chest pain, coughing, vomiting or allergic reactions which require complicated treatment with poor prognosis (post-operative death rate of 2.2% and post-treatment relapse of 6.5%) [1–2].

CE is common in Latin America, where over 2,000 cases are reported annually in Argentina, Brazil, Chile, Uruguay, and Peru [2]. In Chile, echinococcosis has been classified as a mandatory notifiable disease since 2000 [3] and is the leading cause of hospital admissions for parasitic diseases [4–6]. The estimated hospital discharge rate associated with echinococcosis at the national level is estimated at 4.7–5.0 cases per 100,000 population, and approximately 2 cases per 100,000 inhabitants require surgical treatment, mainly affecting the working age group [7–8]. To date, AE has never been reported in Chile [6].

The lifecycle of *E*. *granulosus* is maintained in a dog-livestock-dog cycle. Humans are accidental hosts and are infected via ingestion of embryonated eggs through the environment, food or direct contact with animals [1–2]. Survival of the eggs in the environment, and thus human transmission, is highly impacted by a number of environmental and anthropogenic factors, including climate [9–10]. The presence of large number of dogs harboring the parasite, allowing dogs to feed on uncooked innards or entrails from sheep or cattle, inadequate facilities for slaughter and home slaughtering and consumption have all also been shown to be risk factors for persistence or emergence of CE [7,11–12].

There are currently no official national programs for the control of *E*. *granulosus* in Chile, despite efforts in previous years [8]. Chile first established a CE control program in 1979, which was carried out by the Livestock and Agricultural Service. This program entailed routine deworming of domestic dogs with praziquantel eight times a year in the administrative regions of Aisén and Magallanes (**S1 Fig**). However, due to implementation costs, the frequency of administrating praziquantel was reduced to twice a year. Overall, the program led to a 60–70% reduction in CE prevalence in sheep and dogs over a 27-year period (60% to 0.7% and 71% to 0.5% from 1978 to 2004, respectively) [7–8,13–14]. The program was dismantled in 2004. Subsequently, deworming has been voluntarily carried out by farmers, or through a few local campaigns. Since 2015, four different control programs have been initiated again in the regions of Coquimbo, Bío Bío, Araucanía and Aisén (**S1 Fig**) [7–8,13–14].

In the absence of adequate control programs, there is a risk for re-emergence of CE. Since the national program was terminated in 2004, the change in hospitalization rate for echinococcosis has not been documented. In addition, as Chile starts implementing vaccination programs for CE in animals, surveillance data for CE is important to conclude the impact of its vaccination program in human populations. The objective of our study was to describe the epidemiological trends of hospitalized echinococcosis cases between 2001 and 2012, and investigate the impact of potential risk factors on differences in hospitalization rates between provinces.

## Materials and methods

### Ethics statement

This study involved human subjects and no consent was given. Hospitalization records were deidentified and analyzed anonymously. Patient data could not be linked directly or indirectly to identifiable individuals.

### Data collection

A retrospective study was conducted nation-wide from hospitalized cases diagnosed with CE and reported to the Ministry of Health from January 1st 2001 to December 31st 2012. There are 15 administrative regions in Chile, and hospital discharge data were collected from public and private hospitals in each region (**S1 Fig**). Patients were diagnosed with CE if they had at least one positive imaging test (ultrasound, NMR, CAT or X-ray) and one positive serologic test (in-house IgG ELISA as screening test and in-house IgG, IgM and IgA Western Blot as confirmatory test). Samples were tested at the Public Health Institute of Chile. Cases were coded with the International Classification of Diseases 10th revision (ICD-10) which identified the causative species of echinococcosis and the location of lesions in the body (B670 to B679). Patient information such as age, sex, municipality/region of residence, date of admission, date of discharge, duration of hospitalization and state of discharge were also provided. Livestock, demographic and climate data were obtained from Chile census data [15]. Annual regional population figures were obtained from population census data of Chile, defined as the populations of June 30th of each year [15].

### Data analysis

Demographic and clinical variables were described using proportions for categorical variables and mean or median for continuous variables. Differences in proportions, mean and median were tested with the Chi-squared test, student t-test and Wilcoxon rank test, respectively. Annual regional hospitalization rates (per 100,000) were calculated as the number of CE-related hospitalizations over the total population in the region in a given year. Crude and adjusted annual hospitalization rates were calculated, where rates were sex and age adjusted through direct standardization using the population demographics from 2007 as reference. Livestock density was defined as the number of heads per square kilometer. Annual average temperature was defined as the average temperature during a one-year period in degree Celsius, and total annual precipitation was defined as the total precipitation during a one-year period in millimeters. Trends in hospitalization rates were analyzed by negative binomial regression for the whole study population. Negative binomial regressions were also used to identify correlates of differences in hospitalization rates between provinces for the year 2007 to allow for more input data. Candidate variables were livestock density and climate data from the year 2007 (year at which the livestock census was done) gathered at the provincial level. A total of 11 variables related to climate and animals were evaluated for their association with CE at one point in time. Precipitation was best fit to the data using a generalized linear model with cubic splines to capture non-linear effects. The Akaike Information Criterion (AIC) was calculated for each regression and each combination of input variables (**S1 Table**). We identified 10 models with an AIC score within a difference of two units from the lowest AIC score, indicating similar fits to the model. Ultimately, the most parsimonious model was chosen and included the following three variables: goat density, annual average temperature and annual average precipitation. A log-likelihood ratio test was performed to evaluate the inclusion of precipitation in the final model and revealed statistically significant (p = 0.042). This model was then adjusted for age and sex categories, and the variables remained significant. These variables were used to fit a log-linear regression, to explain the difference in hospitalization rates between the two 2001 and 2007 data points. This time, livestock and climate data from 1997 and 2007 were used. All analyses were conducted using R software version 3.1.3 [16].

## Results

### Descriptive epidemiology

A total of 11,516 hospitalized patients with CE were discharged between 2001 and 2012 (**Table 1**). Slightly more than half of patients were male (52.95%, 6098/11516). The mean age of cases was 40.1 years (standard deviation 21.1). The duration of hospital stay varied between 1 and 292 days with a median of 8 (interquartile range 4–16) days. When classifying the data into age groups based on likely workforce participation, 15.2% (1746/11516) of cases were 0–14 years-old, 65.9% (7586/11516) of cases were 15–59 years-old and 19.0% (2184/11516) were 60 years and above. Demographic data and clinical data are presented in Table 1.

**Table 1:** Characteristics of patients hospitalized for echinococcosis from 2001 to 2012.

	Total	Male	Female	P-value
**Number of cases** n (row %)	11516	6098 (52.95)	5418 (47.05)	<0.001
**Age** mean (SD)	40.06 (21.08)	39.53 (21.03)	40.65 (21.13)	0.004
**Age group** n (column %)	<0.001			
0–14 years	1746 (15.16)	1003 (16.45)	743 (13.71)	<0.001
15–59 years	7433 (64.54)	3903 (64.00)	3530 (65.15)	0.205
60+ years	2337 (20.29)	1192 (19.55)	1145 (21.13)	0.037
**Duration of hospital stay** median, [Interquartile range]	8.00 [4–16]	9.00 [4–17]	8.00 [4–15]	<0.001
**Number discharged alive** n (%)	11415 (99.12)	6032 (98.92)	5383 (99.35)	0.016
**Location of cyst**, n (%)	<0.001			
Liver	5163 (44.83)	2452 (40.21)	2711 (50.04)	<0.001
Lung	851 (7.39)	511 (8.38)	340 (6.28)	<0.001
Bone	17 (0.15)	10(0.16)	7 (0.13)	0.809
Multiple/other location	214 (1.86)	116 (1.90)	98 (1.81)	0.763
Unspecified location	5271 (45.77)	3009 (49.34)	2262 (41.75)	<0.001

When categorizing the locations of cysts by age group, a higher proportion of patients aged 15 years and older had liver cysts (46 to 47%), whereas 34% of patients aged 0–14 years had liver cysts (p<0.01). Conversely, a higher proportion of pediatric cases had lung cysts (12%) compared to patients aged 15 years or older (6 to 7%) (p<0.01) (**S2 Table**).

### Spatial and temporal patterns

The annual hospitalization rate for CE over 12 years was 5.84 per 100,000 populations (**Table 2**). It has been found to be overall steadily decreasing from 2001 (HR = 7.02 per 100,000) to 2012 (HR = 4.53 per 100,000) with a 5% decrease (rate ratio = 0.95 [95% CI: 0.94, 0.96]) calculated by negative binomial model). It has been decreasing for both sex and in all age groups (**Table 2**).

**Table 2:** National hospitalization rate from 2001 to 2012 (per 100,000).

	2001	2002	2003	2004	2005	2006	2007	2008	2009	2010	2011	2012	12-year average
**Total population**	15,401,952	15,668,271	15,837,836	16,001,669	16,165,316	16,332,171	16,504,869	16,686,853	16,876,767	17,066,142	17,255,527	17,444,799	16,436,848
**Crude hospitalization rate**	7.02	7.07	7.67	6.97	6.72	5.92	5.31	4.79	5.02	4.70	4.80	4.53	5.84
Adjusted hospitalization rate*	7.28	7.26	7.76	7.05	6.76	5.92	5.29	4.75	4.97	4.64	4.70	4.45	5.83
**Female and Mal**e													
0–14 years	4.12	3.11	5.47	4.28	4.20	3.33	3.98	2.81	4.25	3.49	2.62	2.96	3.74
15–59 years	7.28	7.69	7.72	6.95	6.65	6.11	5.11	4.81	4.62	4.66	4.77	4.71	5.86
60+ years	13.27	13.12	12.51	13.05	12.39	10.05	8.90	8.26	8.39	6.93	8.46	6.15	9.84
**Female**													
**All ages**	**6.36**	**6.61**	**6.93**	**6.54**	**6.54**	**5.81**	**5.05**	**4.58**	**4.58**	**4.25**	**4.11**	**4.30**	**5.44**
0–14 years	3.16	2.38	4.78	3.52	4.05	2.91	3.48	2.38	3.49	2.91	2.48	3.33	3.25
15–59 years	7.00	7.39	6.95	6.72	6.58	6.06	5.02	4.69	4.25	4.25	4.11	4.56	5.57
60+ years	11.09	11.55	11.18	11.41	10.95	9.66	7.83	7.58	7.74	6.20	6.38	4.51	8.58
**Male**													
**All ages**	**7.54**	**7.54**	**8.43**	**7.41**	**6.9**	**6.03**	**5.57**	**5.00**	**5.47**	**5.16**	**5.51**	**4.77**	**6.24**
0–14 years	5.05	3.81	6.13	5.00	4.33	3.74	4.46	3.22	4.97	4.05	2.75	2.61	4.20
15–59 years	7.56	8.00	8.50	7.18	6.73	6.17	5.20	4.94	5.00	5.07	5.43	4.85	6.16
60+ years	16.14	15.16	14.25	15.16	14.26	10.54	10.27	9.13	9.21	7.85	11.09	8.20	11.44

*Adjusted hospitalization rates were adjusted for age group and sex, by direct standardization based on the age and sex distribution of 2007.

**Fig 1:**
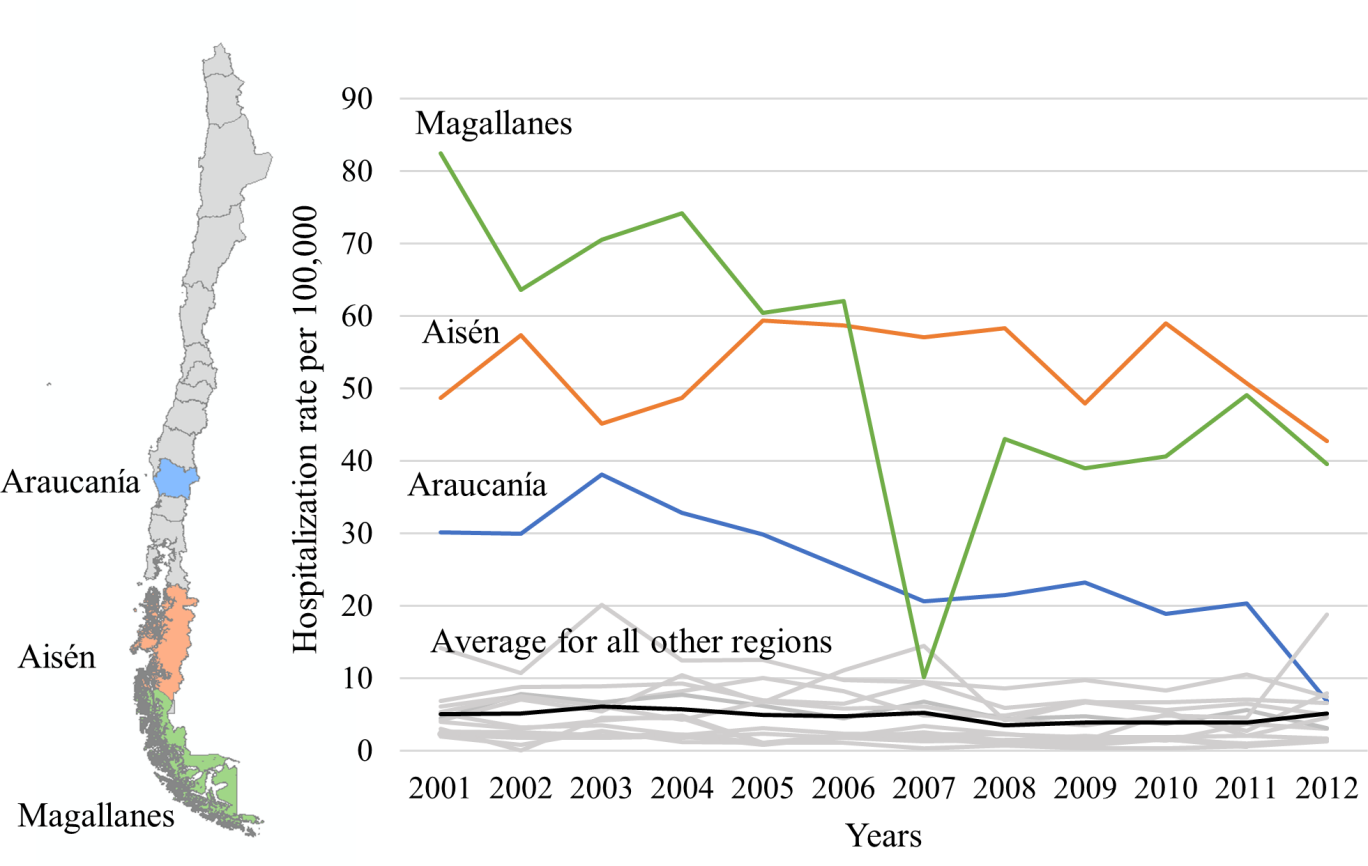
Map and trend of hospitalization rate by region from 2001 to 2012 (per 100,000). The map was created by authors using ArcGIS Desktop (ESRI 2011. ArcGIS Desktop: Release 10.3. Redlands, CA: Environmental Systems Research Institute.) and the base layer was obtained by National Library of Congress of Chile.

The hospitalization rate was consistently higher in the southern regions of Chile (Aisén and Magallanes) and central Chile (Coquimbo) compared to the northern regions of Chile (**Fig 1)**. The hospitalization rate for CE in northern Chile was low over the years, although higher hospitalization rate was observed in some years in the Arica y Parinacota and Los Ríos regions. Temporal and spatial patterns of hospitalization rates are described in Table 2 and in Fig 1.

### Livestock and climate factors

**Fig 2:**
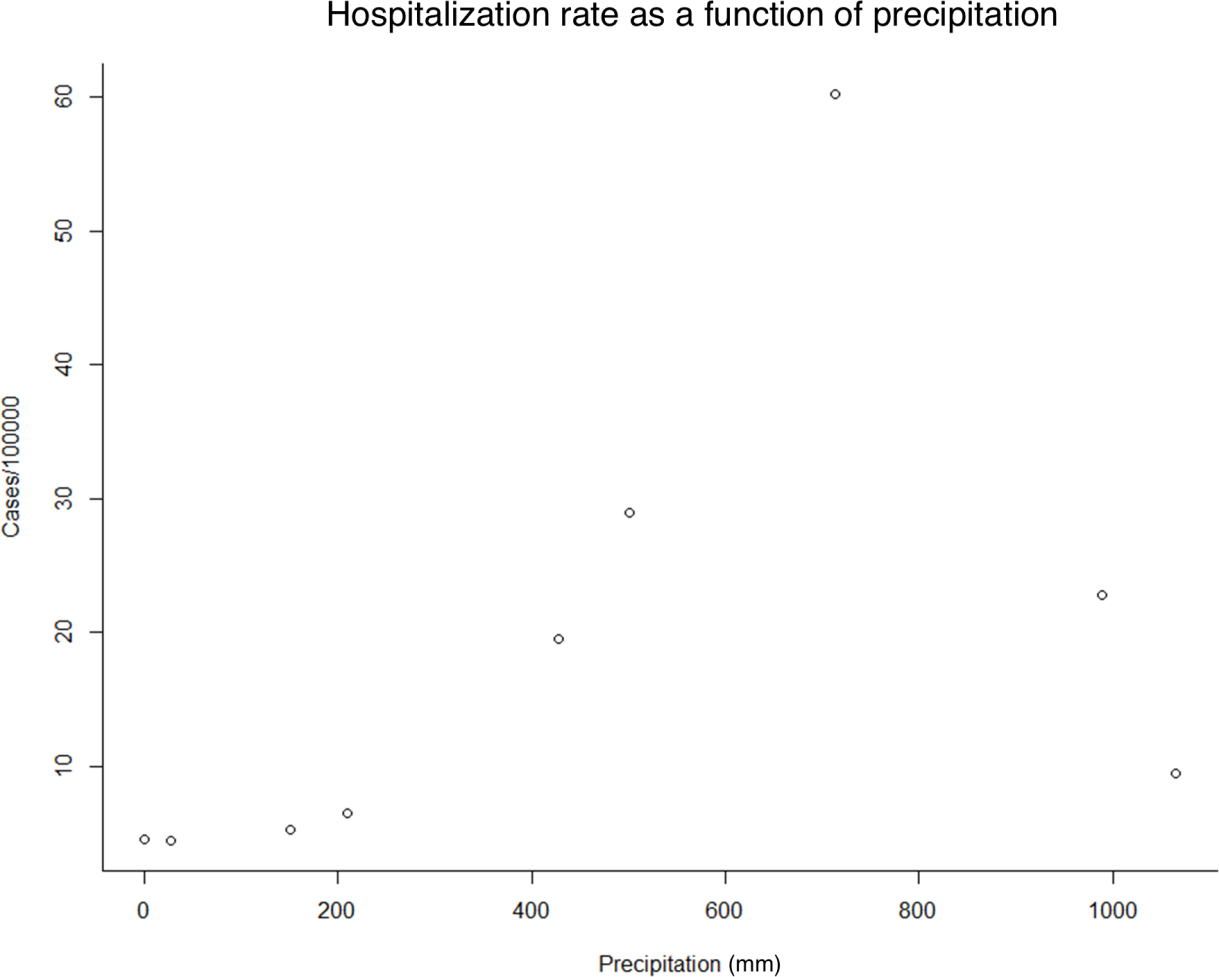
Predicted incidence as a function of precipitation (mm/year). When dividing precipitation into three categories (0≤ to <200, 200≤ to <800, and 800≤), the HRR was calculated as follows, using the lowest category as reference. Precipitation 200≤ to <800 mm/year: 1.60 [0.69,3.81] p-value = 0.21. Precipitation 800≤ mm/year: 1.35 [0.51, 3.69] p-value = 0.52

Goat density was associated with higher hospitalization rates (rate ratio per 10 goats/sqkm = 3.40 [1.58, 7.97]) while annual average temperature was associated with lower hospitalization rates (rate ratio = 0.70 [0.60, 0.82]). Among our observation points, precipitation was significantly associated with higher hospitalization rates for intermediate precipitation levels. The curve of predicted incidence as a function of precipitation is presented in **Fig 2**. Changes in these variables were not predictive of long-term trends in hospitalization rates between 2001 and 2007. Regression results are presented in **Table 3**.

**Table 3:** Risk factors for cystic echinococcosis.

Outcome	Variable	HRR [95%CI] for first model or b[95%CI] for second model
**Regional hospitalization rate**	Goat density (per 10 heads per sq km)	3.40 [1.58,7.97]
	Annual average temperature (°C)	0.70 [0.60, 0.82]
	Annual precipitation (mm)	See **Fig 2**
		*Spline coefficient 1: 0.11[0.007,1.86]*
		*Spline coefficient 2: 17.45[2.20,178.53]*
		*Spline coefficient 3: 0.58[0.14,2.43]*
**Log-Ratio of regional hospitalization rate in 2007/2001**	Goat density ratio	-0.22 [-1.39. 0.95]
	Annual average temperature ratio	2.48 [-1.01, 5.97]
	Annual precipitation ratio (mm)	*Spline coefficient 1: -0.34 [-2.56,1.89]*
		*Spline coefficient 2: -0.06 [-1.85,1.73]*
		*Spline coefficient 3: -0.40 [-0.53,1.34]*

When adjusting for age and sex categories, the impact of each variable on the hospitalization rate stayed similar and did not change our findings (rate ratio per 10 goats/sqkm = 4.11 [2.34, 7.42], rate ratio for annual average temperature = 0.71 [0.64, 0.79] and the predictive curve for annual precipitation was similar and overall statistically significant).

## Discussion

This study is the first to model echinococcosis in Chile, and one of the firsts in Latin America. Although reports on CE prevalence and incidence have previously been reported in Latin American countries, this comprehensive analysis linking human CE cases, host species and environmental factors will provide a basis for approaching CE prevention and control holistically.

This study demonstrated that the hospitalization rates for CE remain high in Chile compared to previous estimates, despite a general decrease over 12 years [7–8]. The majority of cases were found in people of working age, which is consistent with previous findings [5–6]. Although the overall gender difference was small, pediatric cases aged 0 to 14 years were more likely to be male than female (p<0.01). This may be due to behavioral differences between boys and girls, where boys have more exposure to sources of infection such as soil and carcasses. Furthermore, the relationship between patients’ age group and location of cysts suggested that there was a significant difference between the location of the cyst in pediatric and adult cases. Inconsistent findings from previous literature suggest that pediatric cases are more likely to have liver or lung cysts compared to adults, depending on the study [17–18]. In our study, pediatric cases had a high proportion of pulmonary cysts compared to adults, which may be attributed to the slower development rate of liver cysts compared to lung cysts, delaying health seeking behavior for patients with liver cysts in pediatric years [19–20].

The highest hospitalization rates were observed in the southern regions, the highest being in Aisén. This geographical difference between the north and south may be attributed to agricultural characteristics. Southern Chile is highly agricultural, specializing in cattle and sheep husbandry, whereas horticulture and raising camelids are common in the north. It is plausible that southern areas with higher caprine production have higher rates of hospitalization. Another possibility is that hospitalization rates appear higher in rural areas with smaller populations, as one new case may dramatically elevate the rate in the region. In addition to this, we observed a somewhat high hospitalization rate in the Coquimbo region in the central-north part of Chile. One possible explanation is that goat production is common in Coquimbo. This is further supported by our regression model, where higher goat density was significantly associated with a higher hospitalization rate for CE.

We found that the density of goats, temperature and precipitation are important factors that are associated with changes in CE hospitalization rates in Chile. We considered the density of livestock in the model because high density of goats, for example, indicate more intensive goat husbandry, increased risk for dogs to be in contact with infected goat’s viscera, and increased risk for people to be infected by *E*. *granulosus* eggs from dog feces. Goats appear to be an important host for the maintenance and transmission of *Echinococcus granulosus* in Chile. This is somewhat unexpected as sheep are known to be the main risk factor for CE transmission [21–22], but might be explained by the fact that goats are host to more strains of *E*. *granulosus* [23]. Further strain typing data of *Echinococcus* cysts in humans and animals would augment our findings in elucidating the epidemiological link between species. In addition, slaughterhouse data from 2014 indicate a higher cumulative incidence of CE in goats compared to cattle, sheep and horses (305 cases per 1,000 goats, 200 cases per 1,000 cattle, 31 cases per 1,000 sheep; 12 cases per 1,000 horses) [8]. A lower occurrence of CE in sheep and cattle compared to goats may explain why sheep and cattle were not found to be important risk factors in our regression model. Given that people do not become infected with echinococcosis by simply handling or manipulating infected goats or sheep, it is possible that improper handling of goat viscera during and after slaughter increases the risk of dogs, the definitive host, to become infected. This will, in turn, increase people’s risk to become infected through contact with feces of *E*. *granulosus* infected dogs. Although intervention programs in Chile did not target goats in the past, future control measures may be more effective if goats were also targeted.

In addition, lower temperatures and medium range precipitation in Chile are risk factors for CE hospitalizations according to our model. This is consistent with the high hospitalization rate observed in southern Chile, which is characterized with an oceanic climate. It is also supported by previous reports on the survival of the *E*. *granulosus* eggs in the environment [9–10,24]. Low temperatures and humid soil allow for the eggs to survive longer in the environment. Moreover, *E*. *granulosus* eggs are sensitive to high temperatures and low humidity as it leads to loss of infectivity [25]. These are critical factors that increase the risk of infection of livestock and humans and thus the number of hospitalizations. It is also worth noting that areas in Chile with extremely low temperature and low precipitation may also be regions with fewer sheep and goats, since these animals are not able to survive under extreme climates. Changes in climate variables were not predictive of long term hospitalization trends likely due to the time lag between time of infection, presentation of clinical signs and health-care seeking behavior. The association between CE hospitalization rates and the variables that were found to be significant in our model are ecological in nature and cannot be causally interpreted.

The overall decrease in the rate of hospitalizations related to CE could be attributed to national prevention measures and heightened awareness of echinococcosis in Chile since 1979 [13–14]. While large-scale programs at the national level are ideal, resources should be allocated to local, targeted programs in order to decrease transmission. We recommend that the Ministry of Health focus their prevention efforts on farm workers in regions with high hospitalization rates through workshops and other methods of education. Focusing on children’s education in school may be effective in guiding the population to interact with their domestic animals appropriately, including washing hands after grooming them, avoiding kissing them on the mouth, and deworming their dogs every 2 to 3 months. Furthermore, our finding that temperature and precipitation factors are associated with CE hospitalization could be utilized to reinforce targeted prevention measures in areas where climatic conditions are conducive to *E*. *granulosus* survival and transmission or husbandry of intermediate hosts such as sheep and goats. In Chile, this translates to implementing adapted deworming in areas with high concentration of echinococcosis cases, especially in southern Chile and Coquimbo.

Our findings should be interpreted in light of some limitations. First, the outcome that was used to measure CE was the number of hospitalizations. Trend in hospitalization rates does not necessarily reflect trend in disease incidence, but rater hospital seeking behavior in people who are infected with CE, which could be affected by various factors such as severe climate and livestock health. It is also possible for one person to be counted more than once if the patient was hospitalized multiple times for CE. In addition, hospitalization rates may have decreased due to better outpatient care, while the incidence itself stayed constant. However, the ICD-10 coded hospitalization records are the most accurate and consistent data currently available in Chile, and other measures of disease are deemed inaccurate and inconsistent by Chilean public health officials due to significant underreporting. Second, hospitalization rates analyzed by each administrative region and provinces may not perfectly reflect the true incidence of the region. This is because rural dwellers may seek health care in larger cities and the true incidence in rural regions may be higher. Additionally, echinococcosis is a disease developing over the course of several years implying a lag between infection and hospitalization that our model could not take into account. As no data is available on incubation period, we chose to use livestock and climate data from the same hospitalization year as a proxy. Our guess is that the association would be even stronger if we had used data from several years before hospitalization. In order to evaluate the risk of re-emergence of echinococcosis in Chile, targeted studies on high-risk populations such as agricultural workers and veterinarians and their interaction with host animals are required. Studies could investigate the lag between extreme climatic events and epidemics of echinococcosis. Furthermore, examining risk factors in a cohort or case-control study will be beneficial to validate findings from cross-sectional studies on echinococcosis in Latin America. Finally, to confirm the role of livestock and climate variables in hospitalization rates, studies at a smaller geographical scale are recommended for risk analysis.

Improved surveillance will lead to a better understanding of the trends and transmission dynamics of echinococcosis, which will allow for effective prevention and control measures. Although CE is a notifiable disease in Chile, the number of notified cases to the Ministry of Health was much lower than the number of hospitalized cases from 2001 to 2012 (4,288 versus 11,673 cases). The number of hospitalized case is only partially represented by the number of notified cases, which demonstrates the need for collecting data and encouraging laboratories, clinicians and hospitals to report any suspected case of *E*. *granulosus* infection. Raising awareness of echinococcosis among health care workers may be key to increased reporting in the future. Moreover, we recommend that the diagnostic method also be collected as part of patients’ records, since it is currently unavailable and could be useful for future surveillance.

In conclusion, echinococcosis is a zoonosis with a significant public health burden in Chile, related to farming and interaction with livestock, and also environmental conditions such as temperature and precipitation. Long-term efforts should be placed on CE in Chile, ensuring that cases are notified to the Ministry of Health and targeted intervention programs are implemented to reduce the hospitalization rate of the disease.

## Supporting information

S1 Fig Administrative regions of Chile. Fifteen regions listed from north to south. The map was created by authors using ArcGIS Desktop (ESRI 2011. ArcGIS Desktop: Release 10.3. Redlands, CA: Environmental Systems Research Institute) and the base layer was obtained from the National Library of Congress of Chile.
(TIF)

S1 Table AIC calculations. A smaller Akaike Information Criteria (AIC) indicates a better fit.
(XLSX)

S2 Table Location of cysts by age group. (DOCX)
