# Supplementary figures and images for: Trends and correlates of cystic echinococcosis in Chile: 2001–2012
Source: PLoS Negl Trop Dis. 2017 Sep 15;11(9):e0005911. doi: 10.1371/journal.pntd.0005911 (PMC5624646; doi:10.1371/journal.pntd.0005911)

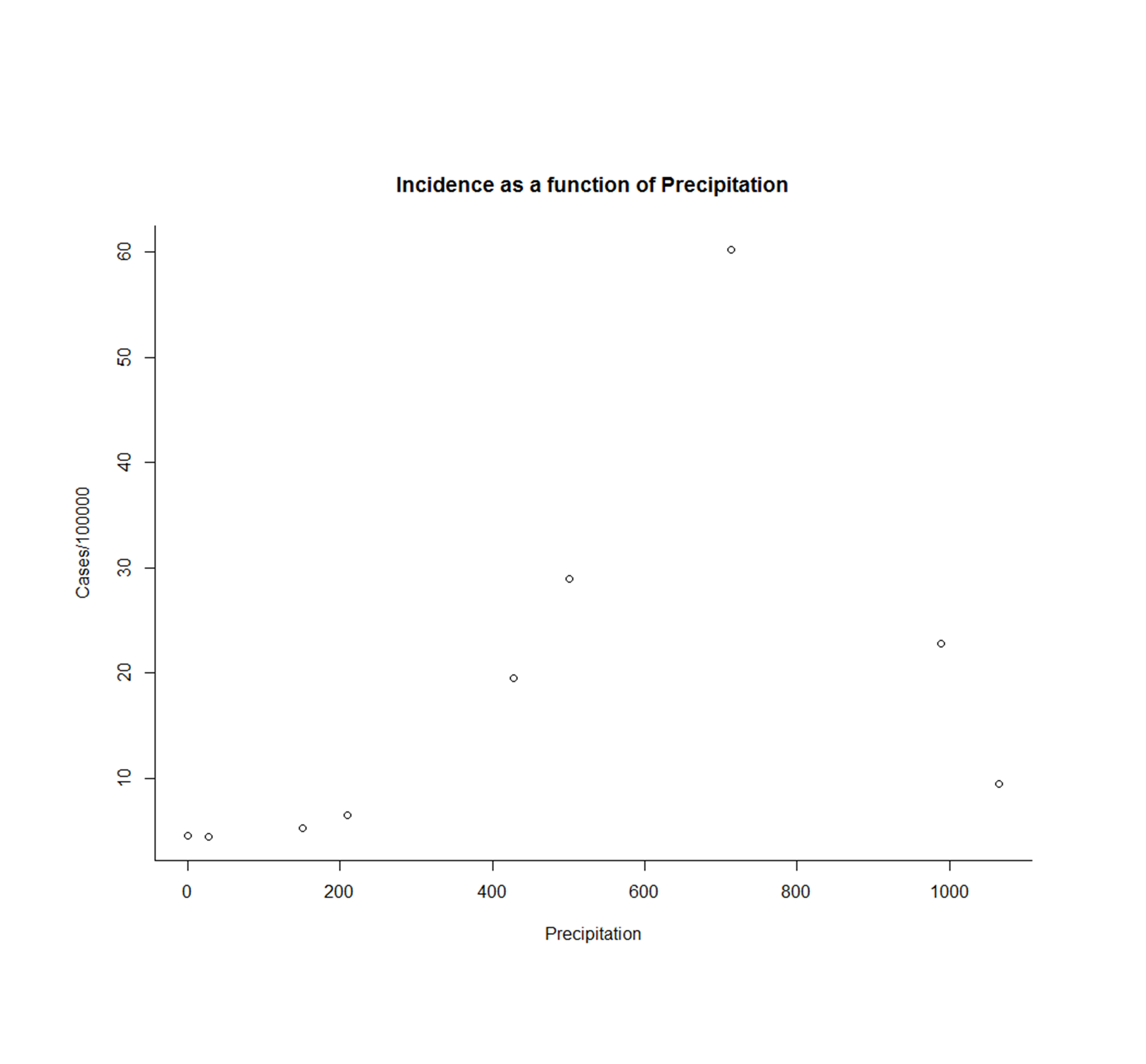

Supplement: S1 Fig — Fifteen regions listed from north to south. The map was created by authors using ArcGIS Desktop (ESRI 2011. ArcGIS Desktop: Release 10.3. Redlands, CA: Environmental Systems Research Institute) and the base layer was obtained from the National Library of Congress of Chile. (TIF) [file pntd.0005911.s001.tif]
