# Supplementary material for: Trends and correlates of cystic echinococcosis in Chile: 2001–2012
Source: PLoS Negl Trop Dis. 2017 Sep 15;11(9):e0005911. doi: 10.1371/journal.pntd.0005911 (PMC5624646; doi:10.1371/journal.pntd.0005911)
Supplement: S2 Table — (DOCX) [file pntd.0005911.s003.docx]

**Table S2. Location of cysts by age group**

|  | **Total**  **n (%)** | **0-14 years**  **n (%)** | **15-59 years**  **n (%)** | **60+ years**  **n (%)** | **P-value*** |
| --- | --- | --- | --- | --- | --- |
| **Liver** | 5163 (44.83) | 594 (34.02) | 3456 (46.50) | 1113 (47.63) | <0.01 |
| **Lung** | 851 (7.39) | 218 (12.49) | 485 (6.52) | 148 (6.33) | <0.01 |
| **Bone** | 17 (0.15) | 2 (0.11) | 11 (0.15) | 4 (0.17) | 0.94 |
| **Multiple/other** | 214 (1.86) | 39 (2.23) | 129 (1.74) | 46 (1.97) | 0.35 |
| **Unspecified** | 5271 (45.77) | 893 (51.15) | 3532 (45.10) | 1026 (43.90) | <0.01 |
| **Total** | 11516 (100.00) | 1746 (100.00) | 7433 (100.00) | 2337 (100.00) | - |

*P-value for chi-square test or fisher’s exact test for 3x2 table for 3 age groups and location (ex: liver vs. non-liver).
